# Akaby—Cell-free protein expression system for linear templates

**DOI:** 10.1371/journal.pone.0266272

**Published:** 2022-04-07

**Authors:** Wakana Sato, Judee Sharon, Christopher Deich, Nathaniel Gaut, Brock Cash, Aaron E. Engelhart, Katarzyna P. Adamala

**Affiliations:** Department of Genetics, Cell Biology and Development, University of Minnesota, Minneapolis, MN, United States of America; Universidad de Jaen, SPAIN

## Abstract

Cell-free protein expression is increasingly becoming popular for biotechnology, biomedical and research applications. Among cell-free systems, the most popular one is based on *Escherichia coli* (*E*. *coli*). Endogenous nucleases in *E*. *coli* cell-free transcription-translation (TXTL) degrade the free ends of DNA, resulting in inefficient protein expression from linear DNA templates. RecBCD is a nuclease complex that plays a major role in nuclease activity in *E*. *coli*, with the RecB subunit possessing the actual nuclease activity. We created a *RecB* knockout of an *E*. *coli* strain optimized for cell-free expression. We named this new strain Akaby. We demonstrated that Akaby TXTL successfully reduced linear DNA degradations, rescuing the protein expression efficiency from the linear DNA templates. The practicality of Akaby for TXTL is an efficient, simple alternative for linear template expression in cell-free reactions. We also use this work as a model protocol for modifying the TXTL source *E*. *coli* strain, enabling the creation of TXTL systems with other custom modifications.

## Introduction

Cell-free transcription-translation (TXTL) has been gathering increased attention in research and industry, due to its versatile application potential in synthetic biology. Those applications include, gene circuit testing [1], artificial cell systems [2–4], protein evolution [5], and enzymatic reaction optimizations [6, 7]. The combination of bacteriophage RNA polymerase T7 and *Escherichia coli* (*E*. *coli*) crude extract is the most popular TXTL system [8]. In TXTL, the proteins are expressed from gene coding DNA in either circular plasmids, or linear DNA fragments. There have been tremendous characterizations and optimizations to improve nearly all aspects of TXTL [1, 9, 10].

Translation of linear DNA templates is of great importance to both research and practical applications. Eliminating the need to clone plasmid DNA, TXTL can be used to significantly increase the speed of the design–build–test cycle in bioengineering. Since the bacterial TXTL degrades linear DNA templates, two nuclease inhibitors are widely used to overcome this problem: GamS or Chi6 (short DNA containing six Chi sites). Neither of these inhibits the nuclease activity completely.

RecBCD is a nuclease complex endogenous to *E*. *coli*. *RecB* contains the nuclease and the helicase domains; *RecC* is implicated to be responsible for Chi sequence recognition; and *RecD* contains the helicase domain [11]. RecBCD plays a vital role in maintaining endogenous genome quality and integrity. One of the most common DNA damage mechanisms is a DNA double-strand break caused by a variety of reasons, such as ionizing radiation or DNA replication errors. To repair this damage, RecBCD binds blunt-ended DNA termini and converts the template into a duplex DNA possessing a 3’-terminated ssDNA tail to initiate homologous recombination. The critical component in this process is the octameric regulatory DNA sequence called Chi (5’-GCTGGTGG-3’) [12, 13]. RecBCD terminates the conversion of dsDNA into ssDNA at the Chi sequence. Since TXTL consists of *E*. *coli* crude extract, endogenous RecBCD remains active in the TXTL reactions. It has been previously demonstrated that the supplementation of Chi6 DNA enhances DNA stability and protein expression in TXTL [14]. This finding strongly suggests the association of RecBCD in *E*. *coli* crude extract with DNA stability and gene expression in TXTL.

Along with the Chi sequence-based DNA degradation, RecBCD also possesses random nuclease activity for blunt-ended DNA fragments. That random nuclease activity initially evolved to defend their own genome from invading DNA, such as lambda and T4 bacteriophages [11]. GamS, a bacteriophage lambda encoded protein, evolaved as a counter-strategy of RecBCD to protect phage DNA by forming a stable complex with RecBCD [15–17] GamS forms a double-stranded DNA mimetic and works as a competitive inhibitor for RecBCD [18]. To protect free-end linear DNA templates in TXTL, GamS has been used as a supplementation of TXTL to increase the protein expression from the gene template of linear PCR fragments [1]. Even though GamS has been used to protect linear DNA fragments in TXTL, it is still unclear which nuclease in TXTL plays the vital role in reducing protein expression from linear templates.

Both GamS and Chi6 DNA protection protocols require adding additional components to TXTL. This not only adds extra steps, costs and failure points to the reaction, but it also uses some of the limited component volume. TXTL reactions are typically set up with very little volume left for additives and templates. We provide a solution to those problems by engineering an *E*. *coli* strain optimized for cell-free TXTL production with inactivated RecBCD nucleases.

It has been previously demonstrated that knocking-out or -down RecBCD nuclease in *E*. *coli* strains result in cell-free lysate that shows improved template stability [19, 20]. We decided to build on that work, using the NEB5α *E*. *coli* strain optimized for TXTL, and creating a strain that will be freely available to the whole community. Using state of the art mutagenesis techniques provides an optimized protocol for future engineering of custom TXTL strains with specific properties.

We engineered the *RecB* knockout *E*. *coli* strain, which we call Akaby. The name was inspired by the Arcade game Pac-Man, where Akabei is the Japanese name for the Blinky ghost nemesis of Pac-Man. Pac-Man figure is commonly used in simplified schemes to indicate nucleases. The Akabei ghost incapacitates Pac-Man, analogous to the Akaby strain with an incapacitated major nuclease.

We demonstrated that RecB is the strongest nuclease affecting unprotected linear DNA fragment stability in TXTL, and Akaby provides protection from linear DNA degradation. Using Akaby cell-free extract for TXTL can be a simple, efficient choice for linear DNA-friendly TXTL platforms.

## Results and discussion

### *RecB* disruption in *E*. *coli*

**Fig 1:**
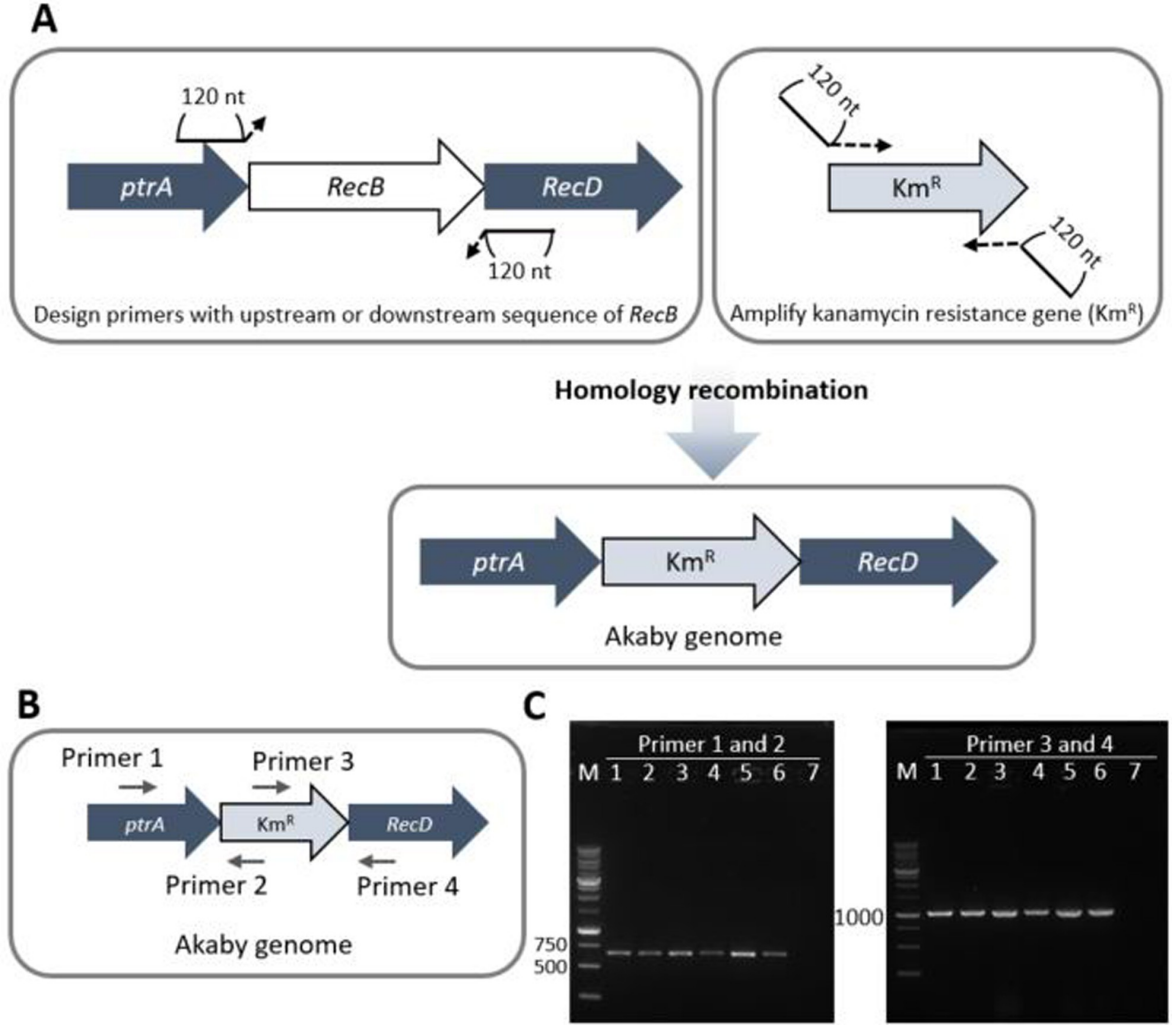
Overview of the *RecB* knockout experiment. (A) The primer designs for Kanamycin resistant gene (Km^R^) PCR. The primers contains 120nt upstream or downstream sequences of *RecB* in *E*. *coli* genome. (B)The primer designs for colony PCR. Primer 1 and 4 have complement sequences outside of *RecB* in *E*. *coli* genome. Primer 2 and 3 have complement sequences within Km^R^. (C) Agarose gel images of colony PCR. 1% agarose gel was run at 125 V for 45min, stained with SYBR safe DNA Gel stain. Expected product size: 661 bp (left gel) and 1035 bp (right gel). Lane M: 1 kb DNA ladder (Goldbio, D010-500) lane 1~6: *RecB* replaced *E*. *coli* NEB5α colonies, 7: wild type *E*. *coli* NEB5α colony.

RecB is the RecBCD subunit that possesses nuclease activity. We deleted the *RecB* gene from the genome of *E*. *coli* by using the λ red system described previously, with minor modifications [21]. The kanamycin-resistant gene (Km^R^) was chosen as a selection marker. On the *E*. *coli* genome, *RecB* is located between *ptrA* and *RecD* (**Fig 1A**). Therefore, we designed primers for Km^R^ PCR amplification with 120 nt homology extensions, which are complementary with the upstream or downstream of *RecB* in *E*. *coli*. These homology extensions promote the replacement of *RecB* with Km^R^ on the *E*. *coli* genome.

After the recombineering procedure, the successful mutant was verified through colony PCR. For the colony PCR, the insert- (Primer 2 and 3) and locus- (Primer 1 and 4) specific primer pairs were used (**Fig 1B**). While no PCR product was detected for the untreated *E*. *coli* colony (**Fig 1C**, lane 7), all six analyzed mutant colonies produced the expected size of PCR products. We thus confirmed that the successful mutant strain that contains the genome replaced *RecB* with Km^R^. We prepared Akaby cell extract from a glycerol stock generated from a single colony among the six confirmed mutants for later experiments.

### Akaby TXTL increased eGFP fluorescence from linear templates

**Fig 2:**
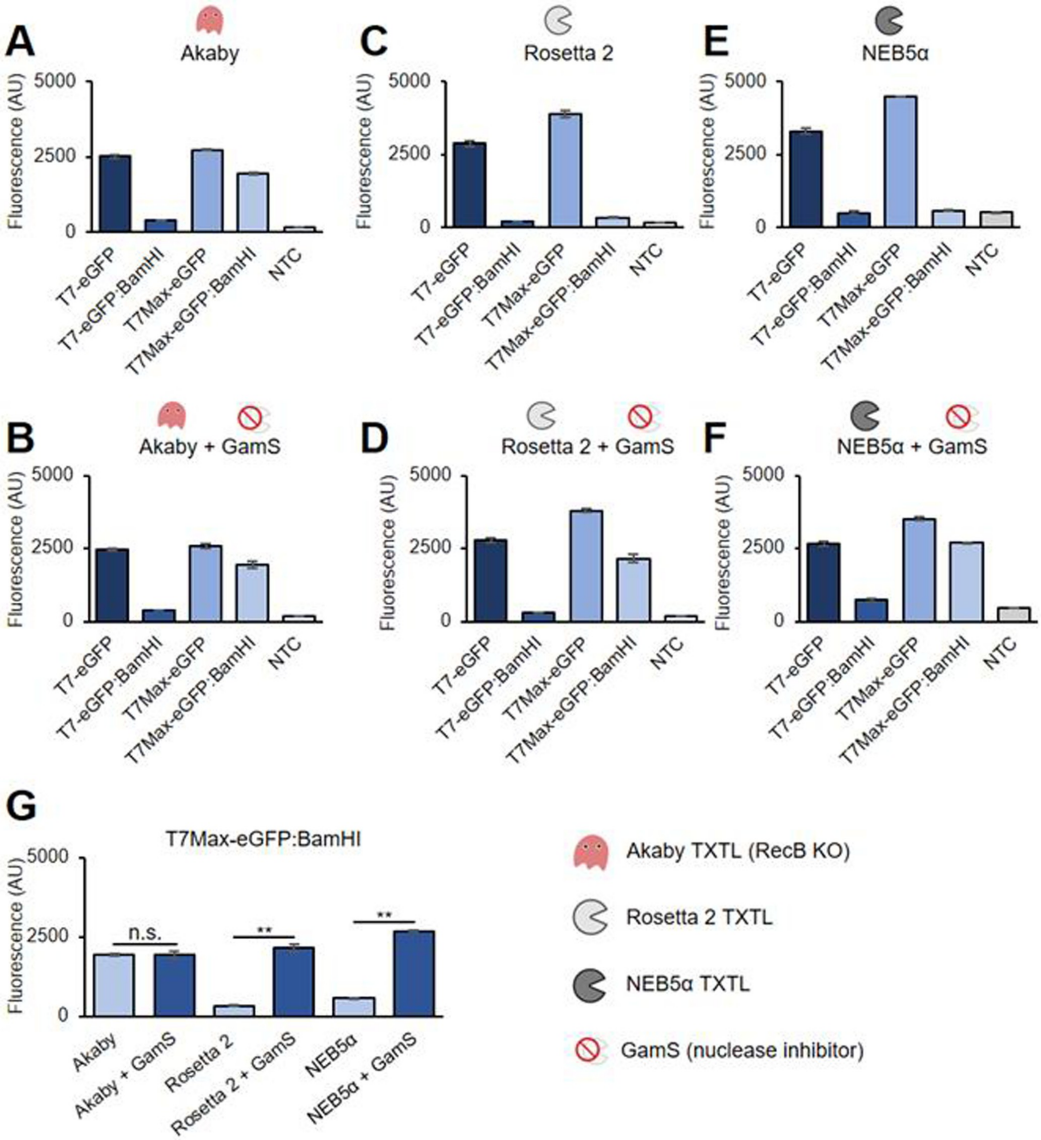
eGFP expression comparison between Akaby, Rosetta 2, and NEB5α TXTL. TXTL reactions were performed with eGFP templates (5 nM) for 8 hours at 30˚C, and each fluorescence was measured at λ_ex_ 488 nm and λ_em_ 509 nm. The eGFP expression in (A) Akaby TXTL, (B) Akaby + Gams TXTL, (C) Rosetta 2 TXTL, (D) Rosetta 2 + GamS TXTL, (E) NEB5α, and (F) NEB5α + GamS. GamS (3.5 μM) was supplemented as a nuclease inhibitor. (G) The TXTL reaction comparison for eGFP fluorescence was generated with the T7Max-eGFP:BamHI template data. T7, the T7 RNA polymerase promoter; T7Max, the enhanced T7 RNA polymerase promoter; template named with BamHI, linearized plasmids by BamHI; NTC, no template control. The graphs show means with error bars that signify SEM (n = 3). Significance was determined by Student’s t-Test. **p < 0.01.

eGFP expressions with circular and linear templates were compared to evaluate the property of Akaby crude extract in cell-free transcription-translation (TXTL) (**Fig 2**). For linear templates, we used eGFP plasmids digested with BamHI. In that plasmid, BamHI cuts at position 182 nt after the eGFP coding sequence, thus linearizing the eGFP plasmid without affecting the open reading frame. To further study the robustness of the system, we used eGFP templates under two different T7 RNA polymerase promoters, the canonical T7 promoter and the new high yield T7Max promoter [22]. Because TXTL components generate a low level of endogenous fluorescence in the green channel, we used a no template control (NTC) as the background fluorescence benchmark. As a comparison to the Akaby cell extract, we used two different *E*. *coli* strains for the cell extract: the Rosetta 2 cell extract that is most commonly used for the TXTL experiments and NEB5α from which Akaby is derived [23].

With the eGFP gene under the regular T7 promoter, we were only able to detect a measurable fluorescence from plasmids. Neither Akaby, Rosetta 2, nor NEB5α TXTL generated a noticeable fluorescence from the linear eGFP templates with the T7 promoter (T7-eGFP:BamHI) (**Fig 2A**, **2C** and **2E**). The linear eGFP templates with the T7Max promoter (T7Max-eGFP:BamHI) generated a fluorescent in Akaby TXTL (**Fig 2A**), while the fluorescence observed in Rosetta 2 and NEB5α TXTL were as low as the NTC (**Fig 2C** and **2E**).

Next, we used the established GamS addition protocol to help protect linear templates in TXTL [1]. We conducted experiments with the supplementation of GamS as a control in parallel (**Fig 2B**, **2D** and **2F**). In Akaby TXTL, GamS supplementation unchanged the fluorescence intensity (**Fig 2G**). On the contrary, in Rosetta 2 and NEB5α TXTL, we could see a significantly higher eGFP fluorescence from T7Max-eGFP:BamHI with the supplementation of GamS (**Fig 2G**). We did not see much fluorescence increase for T7-eGFP:BamHI for all the cell extract with GamS, although there was a slight increase compared to the NTC values (**Fig 2B**, **2D** and **2F**). Previously, GFP expression in TXTL was shown to be saturated above 5 nM for plasmids, while PCR products were above 20 nM with GamS addition [1]. We used 5 nM for all the templates, which might explain why we did not see significant fluorescences from T7-eGFP:BamHI with GamS.

In brief, Akaby TXTL improved protein expression from linear DNA templates, with the difference most apparent under a strong promoter.

### The abundance of mRNA from linear templates are increased in Akaby TXTL

**Fig 3:**
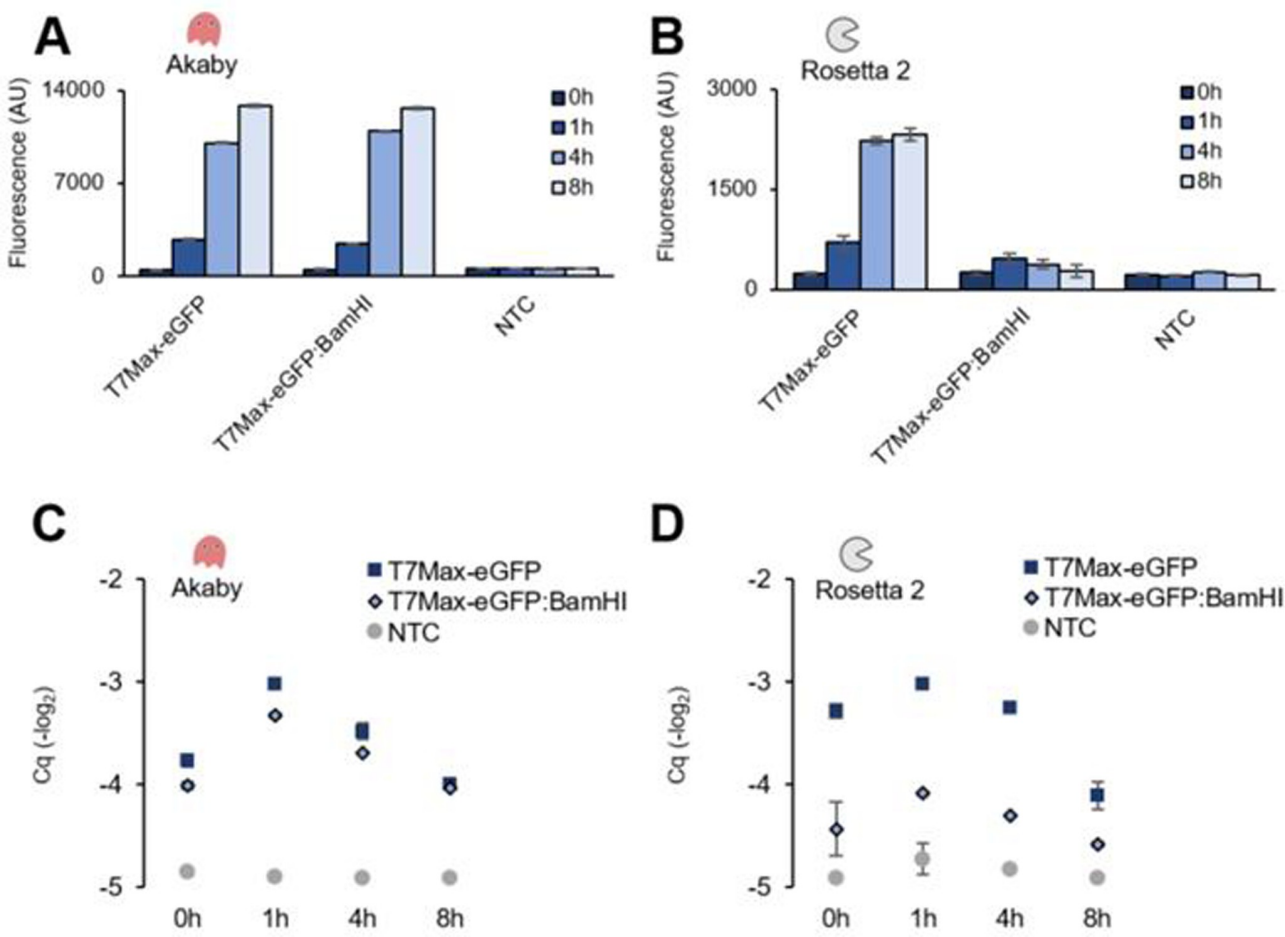
eGFP expression and mRNA abundance in Akaby and Rosetta 2 TXTL at 0, 1, 4, and 8 hours incubation. TXTL reactions were performed with eGFP templates (5 nM) at 30˚C and the fluorescence and mRNA abundance were measured at 0, 1, 4, and 8 hours. The fluorescence was measured at λ_ex_ 488 nm and λ_em_ 509 nm. Fluorescence generated (A) in Akaby TXTL and (B) in Rosetta 2 TXTL. RT-qPCR was performed with a primer pair targeting the eGFP gene. Cq values of mRNA transcribed (C) in Akaby TXTL and (D) in Rosetta 2 TXTL. T7Max, the enhanced T7 RNA polymerase promoter; template named with BamHI, linearized plasmids by BamHI; NTC, no template control; Cq, quantitation cycle. The graphs show means with error bars that signify SEM (n = 3).

The protection of DNA templates from nucleases may increase its transcripts in Akaby TXTL. For further characterization of Akaby TXTL, we measured the amount of mRNA transcribed from eGFP templates and the eGFP fluorescence at several time points: 0, 1, 4, and 8 hours (**Fig 3; S1** and **S2 Figs**).

The fluorescence generated from the template-cell extract pairs was consistent with the result we observed in **Figs 2, 3A** and **3B; S1A**, **S1B**, **S2A** and **S2B Figs**. The quantitative reverse transcription PCR (RT-qPCR) established that mRNA abundance was consistent with the fluorescence measured from eGFP expression reactions (**Fig 3C**, **3D**; **S2C, S2D**, and **S3 Figs**).

Akaby TXTL maintained a relatively high amount of mRNA over 8 hours despite the templates being plasmids or linear fragments (**Fig 3C**). On the contrary, in Rosetta 2 TXTL, while the amounts of mRNA from plasmids were as high as in Akaby TXTL, the mRNA level from linear templates was much lower (**Fig 3D**). The control qPCR on the samples after DNase treatment, which is the procedure before RT-qPCR, ensured that only a negligible amount of DNA remained after DNase treatment **(S3 Fig)**.

These data demonstrated that while Akaby TXTL increased linear template stability, the abundance of mRNA and the fluorescence also increased proportionally.

### Stability of short oligonucleotides without RBS in TXTL improves in Akaby

**Fig 4:**
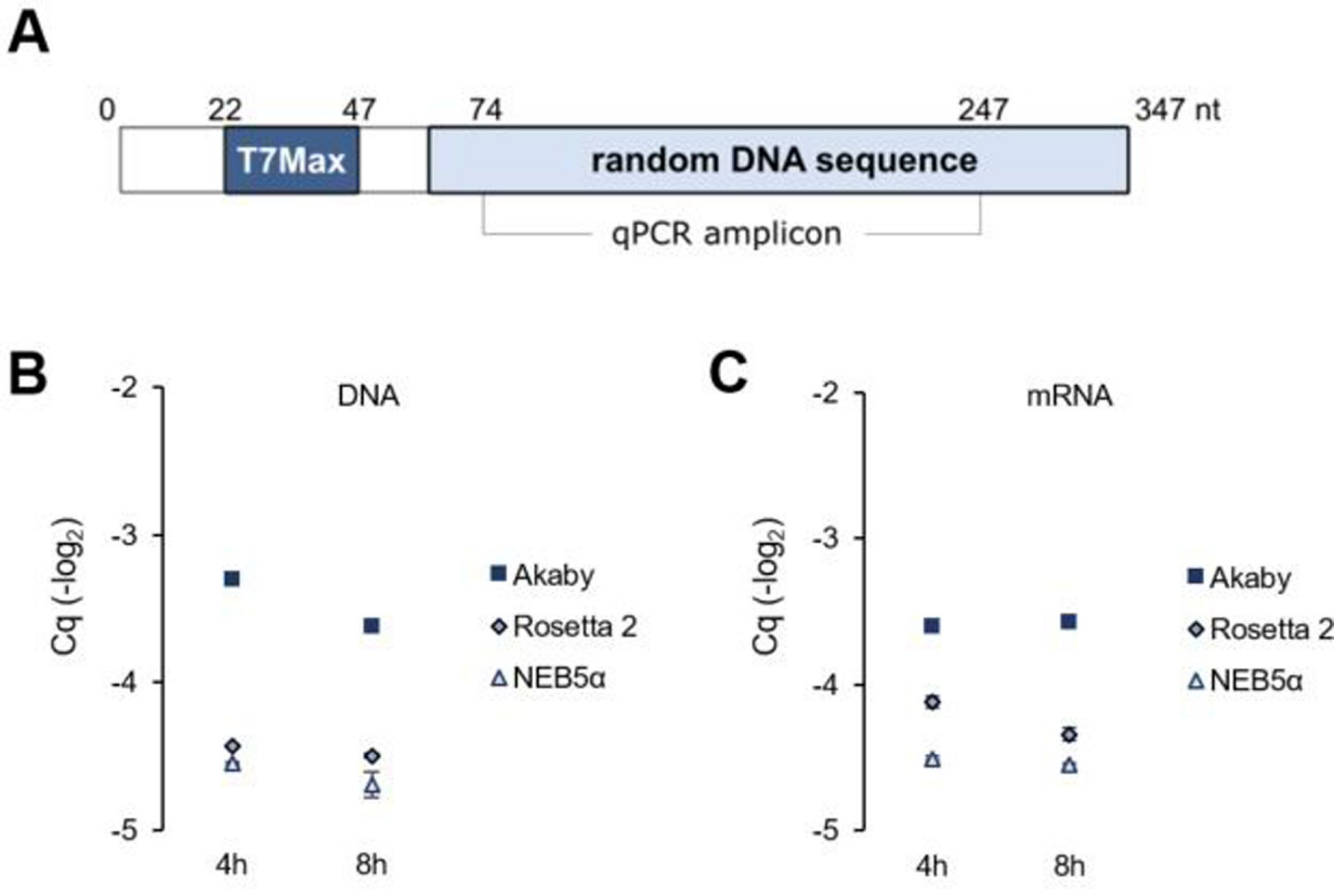
Short DNA stabilities and the abundance of its mRNA in Akaby, Rosetta 2, and NEB5α TXTL at 4 and 8 hours incubation. (A) The design of a 347 nt length DNA fragment. The fragment consists a T7Max promoter and a random DNA sequence. The amplicon for qPCR is as described. (B) The DNA oligo (5 nM) was incubated in Akaby, Rosetta 2, or NEB5α TXTL at 30˚C for 8 hours. After the incubation, DNA was purified with a DNA miniprep kit and qPCR experiments were conducted. (C) The abundances of mRNA in TXTL after 4 and 8 hours incubation were measured by RT-qPCR. T7Max, the enhanced T7 RNA polymerase promoter; Cq, quantitation cycle. The graphs show means with error bars that signify SEM (n = 3).

Since the initially investigated BamHI digested linear GFP DNA template was 3593 nt long, we decided to evaluate the effect of Akaby TXTL on shorter DNA fragments. We designed 347 nt long DNA, consisting of a random sequence under the T7Max promoter. Thus, we can measure the DNA and its transcript stability in TXTL (**Fig 4A**).

We incubated this short linear DNA in Akaby, Rosetta 2, and NEB5α TXTL for 4 and 8 hours, and measured the abundance of DNA and mRNA by qPCR and RT-qPCR accordingly (**Fig 4**).

The DNA fragment and mRNA abundance in Akaby TXTL were the highest among the three different cell extracts (**Fig 4B** and **4C**). Akaby had little effect by GamS supplementation, while GamS supplementation increased the DNA and mRNA abundances in Rosetta 2 and NEB5α TXTL (**S4 Fig**). This observation ensured that Akaby TXTL can also be used for shorter linear DNA fragments.

### Protein expression from PCR amplified products

**Fig 5:**
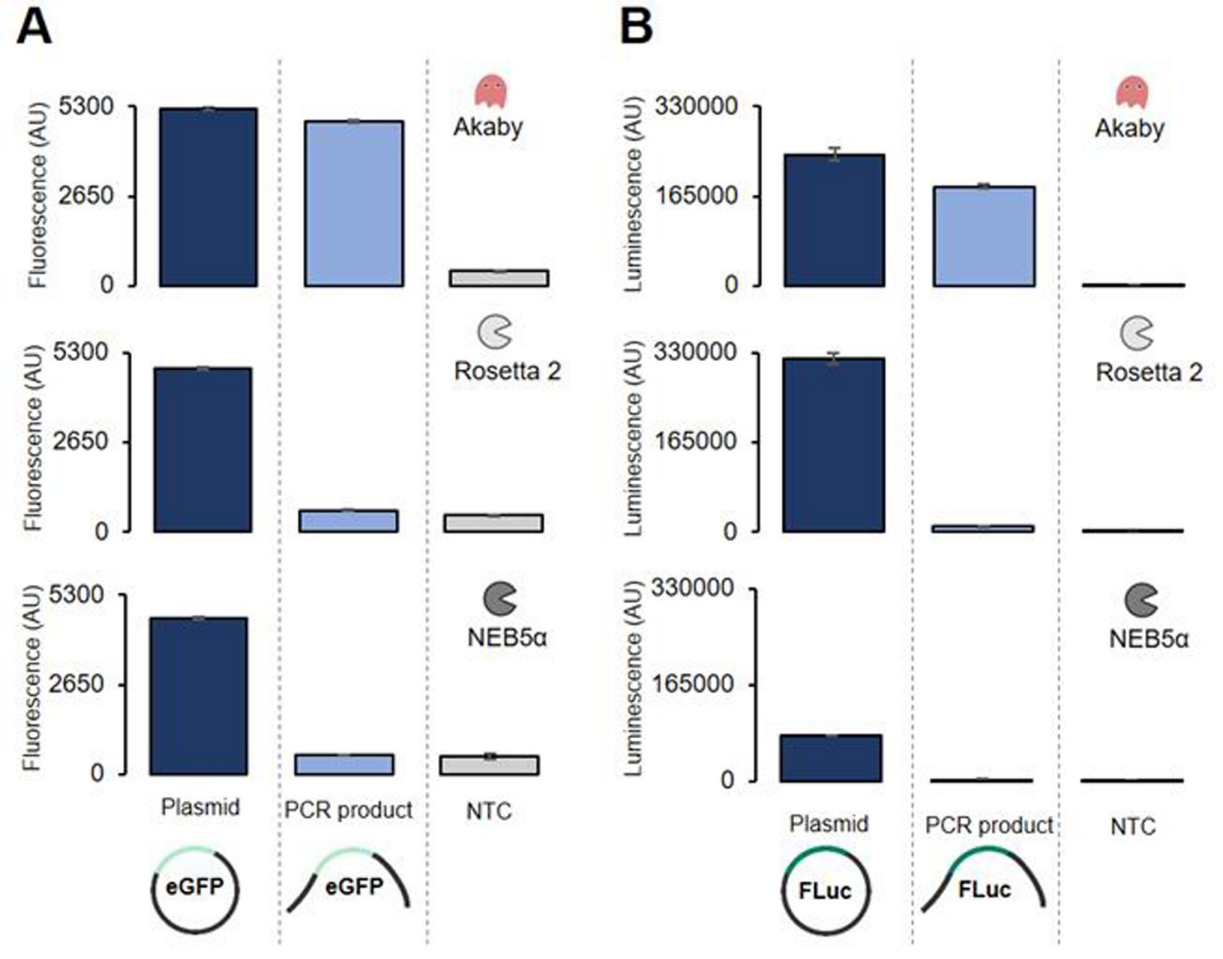
Protein expression from PCR amplified genes in Akaby, Rosetta 2, and NEB5α TXTL. eGFP and firefly luciferase (FLuc) was expressed in Akaby, Rosetta 2, or NEB5α TXTL at 30˚C for 8 hours. The linear PCR product was prepared by PCR amplification from the plasmids. Both the plasmid and the PCR product template concentrations were 5 nM. (A) eGFP fluorescence measured at λ_ex_ 488 nm and λ_em_ 509 nm. (B) The luminescence measurement in FLuc expressing TXTL reactions. After TXTL reaction, 10 μM D-luciferin, 1 mM ATP, and 5 mM MgCl2 were added to the TXTL. The luminescence was measured immediately without emission filters. The graphs show means with error bars that signify SEM (n = 3).

We further tested the Akaby TXTL capacity on PCR amplified genes **(Fig 5)**. eGFP and firefly luciferase (FLuc) genes were PCR amplified from the plasmids and used as the templates for the protein expression in Akaby, Rossetta 2, or NEB5α TXTL. FLuc expression was measured after the TXTL reaction by adding D-luciferin as the substrate. We detected sufficient eGFP and FLuc expressions from the PCR templates in Akaby, while we failed in Rosetta 2 and NEB5α (**Fig 5A** and **5B**). Thus, Akaby is also effective for expressing protein from PCR amplified DNA.

## Discussion and conclusions

We used NEB5α (a DH5α derivative) as a host strain of Akaby, while Rosetta 2 (a BL21 derivatives) is the one most widely used for TXTL [23]. There is a possibility that Rosetta 2 (a BL21 derivative), a protease deficient strain designed for protein overexpression, positively affect TXTL reactions increasing the basal protein expression efficiency. However, we think the basal protein expression differences in this experiment were more likely due to the batch differences for which TXTL is widely known. For example, we used different Akaby cell extract batches on the experiment of **Figs 2A** and **3A**, showing different fluorescent intensities from the identical plasmid (**Figs 2A** and **3A**, T7Max-eGFP data). Since we initially had some experimental difficulties creating a *RecB* knockout mutation with Rosetta 2, we shifted gears to using NEB5α. While we expect to see similar outcomes with Rosetta 2, we decided to leave out for this paper since we already have a working strain for TXTL with linear DNA templates.

In this work, the TXTL extract was prepared using sonication protocol. There is no evidence to suspect any other method of extract preparation, including freeze-thaw, bead beating or French press, should produce Akaby extract with different nuclease properties. We chose sonication for expediency and scalability.

We have observed higher degree of variability in the GamS mediated RecBCD inhibition. The results presented here indicate that GamS inhibition of RecBCD is not always complete, and the effect of GamS is specific to a certain TXTL prep method, the type and length of the DNA template, the concentration of the template and concentration of the GamS protein. Our results, combined with evidence from other papers using GamS for linear template expression, indicate higher degree of variability of results in using the RecBCD inhibition methods.

In summary, we constructed a *RecB* knockout *E*. *coli* strain called Akaby, and we demonstrated a TXTL system based on this strain. Compared to standard TXTL Rosetta 2 extract, Akaby TXTL significantly improved linear DNA stability. Akaby matches the efficiency of GamS protection for linear DNA templates, without the need to purify additional proteins and add more components to the reaction mixture. Akaby TXTL can be used as a versatile TXTL platform for the expression of linear DNA templates.

In addition to the specific use case of linear DNA protection in Akaby, this work demonstrates proof of principle for a pipeline for producing *E*. *coli* TXTL strain mutants. As the use frequency and versatility of TXTL systems increases, this technique can be useful for engineering other *E*. *coli* strains for special TXTL applications.

We will publish information about distribution of the strain on our website, akabycells.org.

## Materials and methods

### *RecB* mutagenesis in *E*. *coli*

The protocol of Datsenko [21] with minor modifications was used for *RecB* disruption. Plasmids were purchased from Addgene. pKD4 (Addgene plasmid # 45605; http://n2t.net/addgene:45605; RRID:Addgene_45605) was used for Km^R^ template [21] and pKDsgRNA-trmI (Addgene plasmid # 89955; http://n2t.net/addgene:89955; RRID:Addgene_89955) was used for the lambda red recombinase system [24]. Primers were purchased from Integrated DNA Technologies, Inc. Km^R^ was PCR amplified with primers (HR FW and RV primer in S1 Table). These primers include 120-nt homology extensions that are complemental sequences of upstream or downstream of *RecB* in *E*. *coli* genome, and 19- or 21-nt priming sequences for pKD4 as a template. After PCR amplification of Km^R^, the reaction mixture was treated with DpnI, and then purified with *GenCatch* PCR Purification Kit (Epoch Life Science, Inc., No. 2360250). pKDsgRNA-trml was transformed into NEB® 5-alpha Competent *E*. *coli* (New England BioLabs Inc., C2987I). The 230 μl of pKDsgRNA-trml carrying *E*. *coli* pre-culture was inoculated in 35 ml SOB and incubated at 30˚C. After 2 hours of incubation with OD_600_~0.2, 150 μl 20% Arabinose was added to the culture to induce the lambda red recombinase system from the pKDsgRNA-trmI plasmid. When the OD_600_ reached 0.45, the cells were treated to be electrically competent. The amplified Km^R^ fragments flanking 120-nt homology arms were transformed into the cells. 1 ml SOC was immediately added after electroporation, and the culture was incubated at 37˚C overnight. The following day, the entire culture was plated on LB plates containing 50 μg/ml Kanamycin, 200 μl culture per plate. The plates were incubated at 37˚C overnight. The six *RecB* disrupted colonies were obtained. 10 μl total volume of colony PCR was performed with 0.4 μM of locus- (Primer 1 and 4) and insert- (Primer 2 and 3) specific primer pairs, 1X OneTaq® Quick-Load® 2X Master Mix with Standard Buffer (New England BioLabs Inc., M0486L), and 2 μl of water suspended colony. The colonies are stored as glycerol stocks, and one of them was used for cell extract preparation.

### Cell extract preparation and TXTL reaction conditions

This protocol was adapted from Noireaux [23] and Jewett [25]. The Rosetta 2 and NEB5α cell extract preparations were followed by the method described previously [26] with one modification. A 750 ml 2xYPTG was grown at 30˚C instead of 37˚C. It was previously described that the lower cultivation temperature improved the protein expression from PCR products, and we needed to compare all the extracts under the same culturing temperature [27]. Akaby extract preparation was performed with the same protocol of Rosetta 2 and NEB5α cell extract preparations with minor modifications. For the starter culture preparation, a 50 ml starter culture of the Akaby was grown to saturation at 30˚C in 2xYPTG with Kanamycin (50 μg/ml). A 750 ml of 2xYPTG culture (without antibiotic) was inoculated with a 10 ml starter culture. The culture was grown at 30˚C to an OD_600_ of 0.4–0.6, then harvested.

Cell-free transcription-translation (TXTL) reaction was performed based on the method described previously [26]. Unless otherwise specified, the template concentration was 5 nM. GamS was added with a concentration of 3.5 μM. The TXTL reaction was incubated at 30˚C for 8 hours, followed by 4˚C temperature hold. For linear eGFP templates preparations, eGFP plasmids were digested with BamHI (New England BioLabs Inc., R0136L) at 37˚C for 1 hour and agarose gel-purified with *GenCatch* Advanced Gel Extraction Kit (Epoch Life Science, Inc., No. 2260050). eGFP fluorescence was measured at λ_ex_ 488 nm and λ_em_ 509 nm with plate reader PMT setting “medium” and 6 reads per well. All fluorescent measurements were performed on SpectraMax. For the endpoint measurement, 14 μl of TXTL reaction was transported into a 384 black bottom well plate to measure after the incubation. For kinetics, 15 μl of TXTL was prepared in a 384 black bottom well plate, sealed with clear sealing tape to avoid evaporation, and measured every hour, including at the start of the incubation of 30˚C.

### Relative comparison of transcripts with Reverse Transcription-quantitative Polymerase Chain Reaction (RT-qPCR)

Template DNA in 2 μl of the TXTL reaction was degraded by adding 0.5 μl of TURBO DNase (2 U/μl, Catalog No. AM2238, Invitrogen). The mixture was incubated at 37˚C for 30 minutes. The enzyme and the expressed proteins were inactivated by adding 15 mM EDTA (Catalog No. E9884, Sigma-Aldrich) at 75˚C for 10 minutes (T100 Thermal Cycler, Bio-Rad). The denatured proteins were pelleted through centrifugation at 3,200 *g* for 2 minutes. For DNA abundance measurement in TXTL, DNA in 20 μl of TXTL reaction was purified with *GenCatch* PCR Purification Kit before reverse transcription, instead of DNase treatment. The DNA fragment binding to the column membrane was eluted with 20 μl water. The elution was re-applied to the column membrane and repeated the elution 2 more times. A 1 μl of the final elution was used for quantitative PCR (qPCR) reaction.

For each protein sample, forward and reverse primers (Integrated DNA Technologies) were created for downstream reverse transcription and qPCR experiments. Each primer pair was compatible with transcripts produced from the regular promoter and T7Max. For eGFP, the forward primer was Primer 5 and the reverse primer was Primer 6. For the shorter DNA fragment, the transcript that was fragmented due to an integrated stop codon in the DNA sequence, forward primer was Primer 7 and the reverse primer was Primer 8.

To prepare the reverse transcription reaction, 2 μl of the DNase-treated sample was mixed with 2 μl of 10 μM reverse primer, 4 μl of 5X Protoscript II Reverse Transcriptase Buffer, 1 μl of Protoscript II Reverse Transcriptase (200 U/μl, Catalog No. M0368, New England BioLabs Inc.), 2 μl of 0.1M dithiothreitol (DTT), 1 μl of 10mM dNTP, 0.2 μl of RNase Inhibitor (Catalog No. M0314, New England BioLabs Inc.), and 8 μl of nuclease-free water. The reverse transcription reaction was incubated at 42˚C for 1 hour and the reverse transcriptase was inactivated at 65˚C for 20 minutes.

The qPCR reaction mix was prepared by mixing 1 μl of complementary DNA from the reverse transcription with 2 μl of 10 μM forward and reverse primers, 11.25 μl OneTaq Hot Start 2X Master Mix with Standard Buffer (Catalog No. M0484, New England BioLabs Inc.), 1.25 μl Chai Green Dye 20X (Catalog No. R01200, Chai Bio), and 7.5 μl of nuclease-free water. The RT-qPCR was completed using Open qPCR (Chai Biotechnologies) with the following thermocycling program: 1 cycle of 30 second denaturation at 95˚C, 30 cycles of 15 second denaturation at 95˚C, 15 second annealing at 50˚C, 1 minute extension at 68˚C, and 1 cycle of 5 minutes final extension at 68˚C. The amplification curves plotted through the Open qPCR software to determine Cq values. The averages across 3 replicates of each promoter type were calculated separately, replacing non-detected value with 30 (the maximam cycle number).

### Short DNA fragment preparation

The oligo template and primers (Primer 9 and Primer 10) for the amplification (Sequence is in S1 Table) were purchased from Integrated DNA Technologies, Inc. The oligo was PCR amplified with OneTaq® 2X Master Mix with Standard Buffer (New England BioLabs Inc., M0482L), followed by agarose gel purification with *GenCatch* Advanced Gel Extraction Kit (Epoch Life Science, Inc., No. 2260050).

### PCR amplified eGFP and FLuc expression

The eGFP and firefly luciferase (FLuc) genes were amplified with Q5 High-Fidelity DNA Polymerase (NEB, M0491L), primer 11 and primer 12 (S1 Table) from plasmids T7Max-eGFP or T7Max-FLuc (S2 Table), respectively. The PCR products were agarose gel purified with *GenCatch* Advanced Gel Extraction Kit (Epoch Life Science, Inc., No. 2260050). The 5 nM PCR amplified templates or the plasmids were incubated in TXTL reaction at 30˚C for 8 hours, followed by 4˚C temperature hold. The fluorescence of 14 μl of eGFP expressing TXTL was measured at λ_ex_ 488 nm and λ_em_ 509 nm with plate reader PMT setting “medium” and 6 reads per well. For luminescence measurement, 10 μl of FLuc expressing TXTL was mixed with 10 μM D-luciferin, 5 mM MgCl_2_, 1mM ATP, and water to be the total volume of 25 μl reaction mixture. The luminescence was measured in 384 well white flat bottom assay plates (Corning®, 3705) immediately after mixing the reagent by a plate reader (SpectraMax) without emission filters, with PMT setting “medium” and 6 reads per well.

## Supporting information

S1 Fig eGFP expression and mRNA abundance in Akaby and Rosetta 2 TXTL with T7 promoter templates. Fluorescence and mRNA abundance were measured at 0, 1, 4, and 8 hours. The eGFP fluorescence was measured at λex 488 nm and λem 509 nm. Fluorescence generated (A) in Akaby TXTL and (B) in Rosetta 2 TXTL. RT-qPCR was performed with a primer pair targeting the eGFP gene. Cq values of mRNA (C) in Akaby TXTL and (D) in Rosetta 2 TXTL. T7, the T7 RNA polymerase promoter; template named with BamHI, linearized plasmids by BamHI; NTC, no template control; Cq, quantitation cycle. The graphs show means with error bars that signify SEM (n = 3).
(DOCX)

S2 Fig eGFP expression and mRNA abundance in Akaby and Rosetta 2 TXTL with GamS supplementation. Fluorescence and mRNA abundance were measured at 0, 1, 4, and 8 hours. The TXTL reactions were incubated in a black-bottom well plate in a plate reader measuring at λex 488 nm and λem 509 nm. Fluorescence generated (A) in Akaby + GamS TXTL and (B) in Rosetta 2 + GamS TXTL. RT-qPCR was performed with a primer pair targeting the eGFP gene. The Cq values of mRNA transcribed (C) in Akaby + GamS TXTL and (D) in Rosetta 2 + GamS TXTL. T7, the T7 RNA polymerase promoter; T7Max, the enhanced T7 RNA polymerase promoter; template name with BamHI, linearized plasmids by BamHI; Cq, quantitation cycle. The graphs show means with error bars that signify SEM (n = 3).
(DOCX)

S3 Fig DNA abundance after DNAse treatment. qPCR was performed on samples after DNase treatment without reverse transcription. The samples used were Akaby TXTL, expressing eGFP from T7Max-eGFP or T7Max-eGFP:BamHI, at 0, 1, 4, and 8h incubations. qPCR was performed with a primer pair targeting the eGFP gene. T7Max, the enhanced T7 RNA polymerase promoter; eGFP, enhanced green fluorescence protein gene; NTC, no template control; Cq quantitation cycle.
(DOCX)

S4 Fig The short DNA fragment stability and its mRNA abundance with GamS supplementation. The short DNA fragments in Fig 4A were incubated in TXTL with GamS supplementation. (Left) DNA in the TXTL was purified with a miniprep kit and qPCR was performed. (Right) mRNA abundance was measured by RT-qPCR. TXTL samples was DNase treated and then reverse transcribed, followed by qPCR measurement.
(DOCX)

S5 Fig DNA abundance after DNase treatment in the short DNA fragment stability test. qPCR was performed on samples after DNase treatment without reverse transcription. The samples used were NEB5α or NEB5α+GamS TXTL, containing short DNA fragment in Fig 4A. The TXTL was incubated for 4 or 8 hours before qPCR procedure. qPCR was performed with a primer pair targeting the short DNA fragment described in Fig 4A.
(DOCX)

S1 Table Oligonucleotide sequences. (DOCX)

S2 Table Plasmid information. (DOCX)

S1 Raw images (PDF)

## Abbreviations

TXTL: cell-free transcription-translation
eGFP: enhanced green fluorescent protein
FLuc: firefly luciferase
RT-qPCR: quantitative reverse transcription PCR
