# Supplementary figures and images for: Akaby—Cell-free protein expression system for linear templates
Source: PLoS One. 2022 Apr 7;17(4):e0266272. doi: 10.1371/journal.pone.0266272 (PMC8989226; doi:10.1371/journal.pone.0266272)

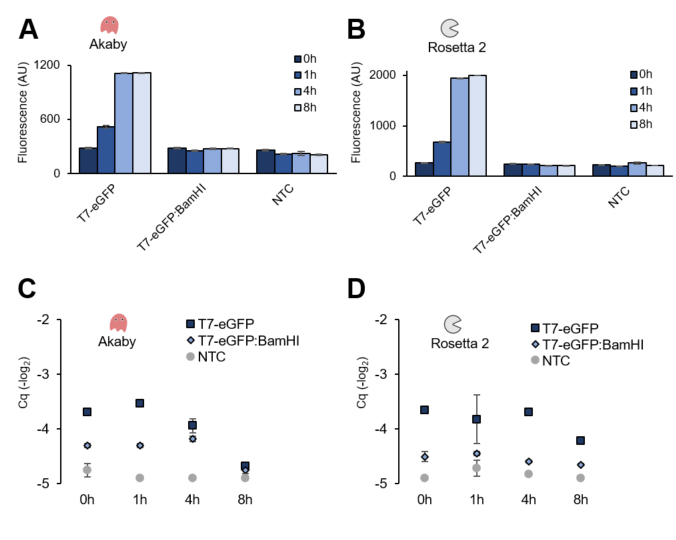


**SFig. 1 eGFP expression and mRNA abundance in Akaby and Rosetta 2 TXTL with T7 promoter templates.**

Supplement: S1 Fig — Fluorescence and mRNA abundance were measured at 0, 1, 4, and 8 hours. The eGFP fluorescence was measured at λex 488 nm and λem 509 nm. Fluorescence generated (A) in Akaby TXTL and (B) in Rosetta 2 TXTL. RT-qPCR was performed with a primer pair targeting the eGFP gene. Cq values of mRNA (C) in Akaby TXTL and (D) in Rosetta 2 TXTL. T7, the T7 RNA polymerase promoter; template named with BamHI, linearized plasmids by BamHI; NTC, no template control; Cq, quantitation cycle. The graphs show means with error bars that signify SEM (n = 3). (DOCX) [file pone.0266272.s001.docx]

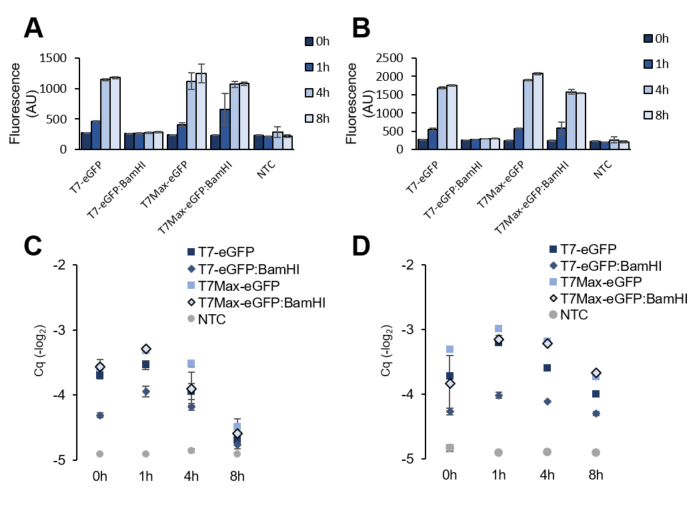


**SFig. 2** **eGFP expression and mRNA abundance in Akaby and Rosetta 2 TXTL with GamS supplementation.**

Supplement: S2 Fig — Fluorescence and mRNA abundance were measured at 0, 1, 4, and 8 hours. The TXTL reactions were incubated in a black-bottom well plate in a plate reader measuring at λex 488 nm and λem 509 nm. Fluorescence generated (A) in Akaby + GamS TXTL and (B) in Rosetta 2 + GamS TXTL. RT-qPCR was performed with a primer pair targeting the eGFP gene. The Cq values of mRNA transcribed (C) in Akaby + GamS TXTL and (D) in Rosetta 2 + GamS TXTL. T7, the T7 RNA polymerase promoter; T7Max, the enhanced T7 RNA polymerase promoter; template name with BamHI, linearized plasmids by BamHI; Cq, quantitation cycle. The graphs show means with error bars that signify SEM (n = 3). (DOCX) [file pone.0266272.s002.docx]

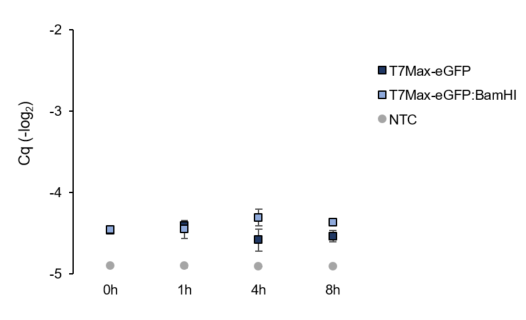


**SFig. 3 DNA abundance after DNAse treatment.**

Supplement: S3 Fig — qPCR was performed on samples after DNase treatment without reverse transcription. The samples used were Akaby TXTL, expressing eGFP from T7Max-eGFP or T7Max-eGFP:BamHI, at 0, 1, 4, and 8h incubations. qPCR was performed with a primer pair targeting the eGFP gene. T7Max, the enhanced T7 RNA polymerase promoter; eGFP, enhanced green fluorescence protein gene; NTC, no template control; Cq quantitation cycle. (DOCX) [file pone.0266272.s003.docx]

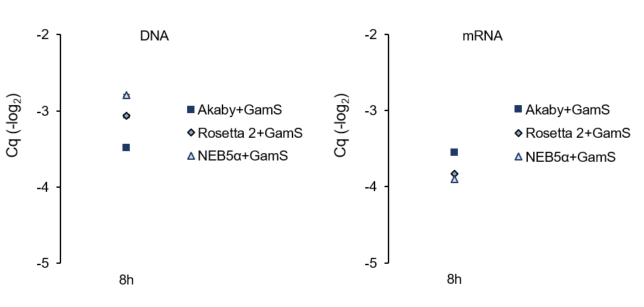


**SFig. 4 The short DNA fragment stability and its mRNA abundance with GamS supplementation.**

Supplement: S4 Fig — The short DNA fragments in Fig 4A were incubated in TXTL with GamS supplementation. (Left) DNA in the TXTL was purified with a miniprep kit and qPCR was performed. (Right) mRNA abundance was measured by RT-qPCR. TXTL samples was DNase treated and then reverse transcribed, followed by qPCR measurement. (DOCX) [file pone.0266272.s004.docx]

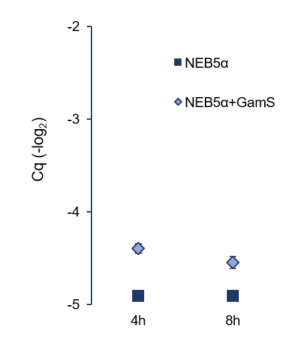


**SFig. 5 DNA abundance after DNase treatment in the short DNA fragment stability test.**

Supplement: S5 Fig — qPCR was performed on samples after DNase treatment without reverse transcription. The samples used were NEB5α or NEB5α+GamS TXTL, containing short DNA fragment in Fig 4A. The TXTL was incubated for 4 or 8 hours before qPCR procedure. qPCR was performed with a primer pair targeting the short DNA fragment described in Fig 4A. (DOCX) [file pone.0266272.s005.docx]

All lanes of this gel were used on Figure 1c

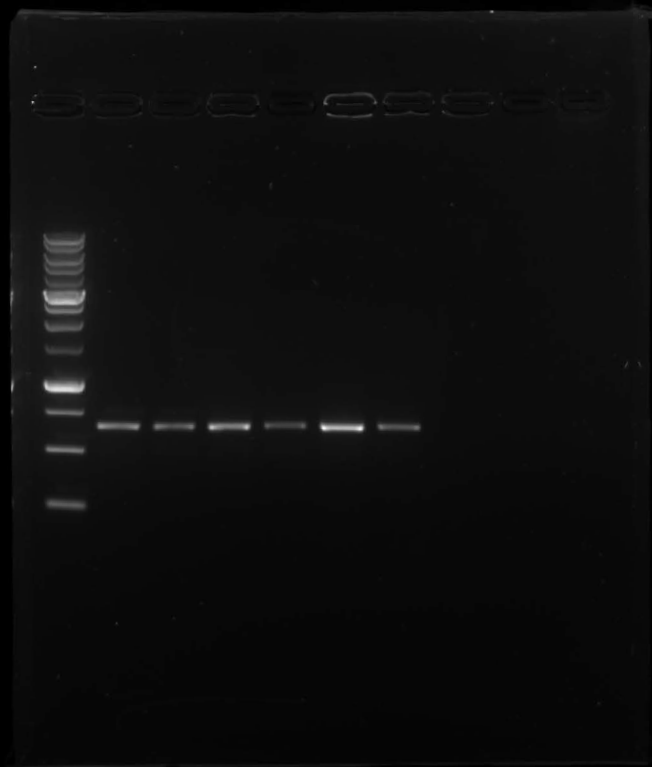

All lanes of this gel were used  
on figure 1c

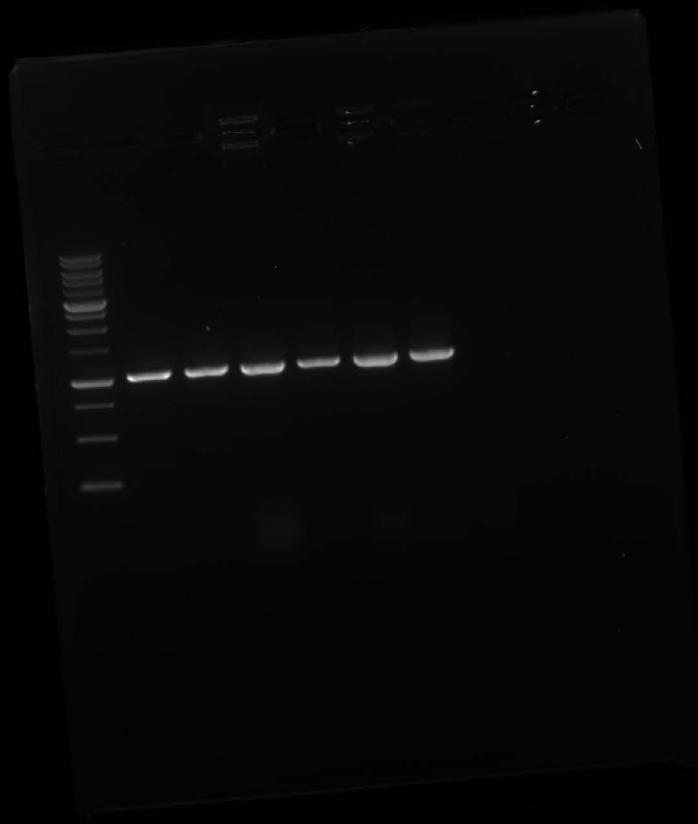

Supplement: S1 Raw images — (PDF) [file pone.0266272.s008.pdf]
