# Supplementary material for: Akaby—Cell-free protein expression system for linear templates
Source: PLoS One. 2022 Apr 7;17(4):e0266272. doi: 10.1371/journal.pone.0266272 (PMC8989226; doi:10.1371/journal.pone.0266272)
Supplement: S1 Table — (DOCX) [file pone.0266272.s006.docx]

**(STable 1) Oligonucleotide sequences**

| Name | 5'-3' oligonucleotide sequence | Purpose |
| --- | --- | --- |
| HR FW primer | TTCTGTCGCAGATTTCCGGCAGCCAGAACGGGAAAGCCGAATATGTACACCCTGAAGGCTGGAAAGTGTGGGAGAACGTCAGCGCGTTGCAGCAAACAATGCCCCTGATGAGTGAAAAGAGTGTAGGCTGGAGCTGCTTCG | RecB knockout |
| HR RV primer | GTGCCGCCGCGAGGGTGACGGCAGGATGTTCATCTCCCGCCACGGTCAGGGCAAATTGCACATCCAGCGGGCGTAGCTGTTTGTGCTCCACAGCTTCCAGTAATTGCTTTTGCAATTTCACATATGAATATCCTCCTTA | RecB knockout |
| Primer 1 | ATTTCTTCCATCAGGCGGT | Colony PCR |
| Primer 2 | CAGTCATAGCCGAATAGCCT | Colony PCR |
| Primer 3 | CGGTGCCCTGAATGAACTGC | Colony PCR |
| Primer 4 | GTCCCTCTCCGGCATCATGA | Colony PCR |
| Primer 5 | AAGTTCATCTGCACCACC | RT-qPCR |
| Primer 6 | TTGAAGTCGATGCCCTTC | RT-qPCR |
| Primer 7 | AAACGGCCACAAGTTCAGC | RT-qPCR |
| Primer 8 | GCTTCATGTGGTCGGGGTA | RT-qPCR |
| Oligo template | CTCACATGGCTCGACAGATCTAATTCTAATACGACTCACTATAGGGAATCCTGGTCGAGCTGGACGGCGACGTAAACGGCCACAAGTTCAGCGTGTCCGGCGAGGGCGAGGGCGATGCCACCTACGGCAAGCTGACCCTGAAGTTCATCTGCACCACCGGCAAGCTGCCCGTGCCCTGGCCCACCCTCGTGACCACCCTGACCTACGGCGTGCAGTGCTTCAGCCGCTACCCCGACCACATGAAGCAGCACGACTTCTTCAAGTCCGCCATGCCCGAAGGCTACGTCCAGGAGCGCACCATCTTCTTCAAGGACGACGGCAACTACAAGACCCGCGCCGAGGTGAAG | Short oligo stability in TXTL |
| Primer 9 | CTCACATGGCTCGACAGATCTAATTC | Short oligo stability in TXTL |
| Primer 10 | CTTCACCTCGGCGCG | Short oligo stability in TXTL |
| Primer 11 | CCGCTTTTTTGCACAACATGG | PCR for eGFP and FLuc gene |
| Primer 12 | TCCTCCGATCGTTGTCAGAA | PCR for eGFP and FLuc gene |
| T7Max promoter | AATTCTAATACGACTCACTATAGGGA | GFP expression in TXTL |
| eGFP gene | ATGGAGCTTTTCACTGGCGTTGTTCCCATCCTGGTCGAGCTGGACGGCGACGTAAACGGCCACAAGTTCAGCGTGTCCGGCGAGGGCGAGGGCGATGCCACCTACGGCAAGCTGACCCTGAAGTTCATCTGCACCACCGGCAAGCTGCCCGTGCCCTGGCCCACCCTCGTGACCACCCTGACCTACGGCGTGCAGTGCTTCAGCCGCTACCCCGACCACATGAAGCAGCACGACTTCTTCAAGTCCGCCATGCCCGAAGGCTACGTCCAGGAGCGCACCATCTTCTTCAAGGACGACGGCAACTACAAGACCCGCGCCGAGGTGAAGTTCGAGGGCGACACCCTGGTGAACCGCATCGAGCTGAAGGGCATCGACTTCAAGGAGGACGGCAACATCCTGGGGCACAAGCTGGAGTACAACTACAACAGCCACAACGTCTATATCATGGCCGACAAGCAGAAGAACGGCATCAAGGTGAACTTCAAGATCCGCCACAACATCGAGGACGGCAGCGTGCAGCTCGCCGACCACTACCAGCAGAACACCCCCATCGGCGACGGCCCCGTGCTGCTGCCCGACAACCACTACCTGAGCACCCAGTCCGCCCTGAGCAAAGACCCCAACGAGAAGCGCGATCACATGGTCCTGCTGGAGTTCGTGACCGCCGCCGGGATC | GFP expression in TXTL |
| FLuc gene | ATGGAAGACGCCAAAAACATAAAGAAAGGCCCGGCGCCATTCTATCCGCTGGAAGATGGAACCGCTGGAGAGCAACTGCATAAGGCTATGAAGAGATACGCCCTGGTTCCTGGAACAATTGCTTTTACAGATGCACATATCGAGGTGGACATCACTTACGCTGAGTACTTCGAAATGTCCGTTCGGTTGGCAGAAGCTATGAAACGATATGGGCTGAATACAAATCACAGAATCGTCGTATGCAGTGAAAACTCTCTTCAATTCTTTATGCCGGTGTTGGGCGCGTTATTTATCGGAGTTGCAGTTGCGCCCGCGAACGACATTTATAATGAACGTGAATTGCTCAACAGTATGGGCATTTCGCAGCCTACCGTGGTGTTCGTTTCCAAAAAGGGGTTGCAAAAAATTTTGAACGTGCAAAAAAAGCTCCCAATCATCCAAAAAATTATTATCATGGATTCTAAAACGGATTACCAGGGATTTCAGTCGATGTACACGTTCGTCACATCTCATCTACCTCCCGGTTTTAATGAATACGATTTTGTGCCAGAGTCCTTCGATAGGGACAAGACAATTGCACTGATCATGAACTCCTCTGGATCTACTGGTCTGCCTAAAGGTGTCGCTCTGCCTCATAGAACTGCCTGCGTGAGATTCTCGCATGCCAGAGATCCTATTTTTGGCAATCAAATCATTCCGGATACTGCGATTTTAAGTGTTGTTCCATTCCATCACGGTTTTGGAATGTTTACTACACTCGGATATTTGATATGTGGATTTCGAGTCGTCTTAATGTATAGATTTGAAGAAGAGCTGTTTCTGAGGAGCCTTCAGGATTACAAGATTCAAAGTGCGCTGCTGGTGCCAACCCTATTCTCCTTCTTCGCCAAAAGCACTCTGATTGACAAATACGATTTATCTAATTTACACGAAATTGCTTCTGGTGGCGCTCCCCTCTCTAAGGAAGTCGGGGAAGCGGTTGCCAAGAGGTTCCATCTGCCAGGTATCAGGCAAGGATATGGGCTCACTGAGACTACATCAGCTATTCTGATTACACCCGAGGGGGATGATAAACCGGGCGCGGTCGGTAAAGTTGTTCCATTTTTTGAAGCGAAGGTTGTGGATCTGGATACCGGGAAAACGCTGGGCGTTAATCAAAGAGGCGAACTGTGTGTGAGAGGTCCTATGATTATGTCCGGTTATGTAAACAATCCGGAAGCGACCAACGCCTTGATTGACAAGGATGGATGGCTACATTCTGGAGACATAGCTTACTGGGACGAAGACGAACACTTCTTCATCGTTGACCGCCTGAAGTCTCTGATTAAGTACAAAGGCTATCAGGTGGCTCCCGCTGAATTGGAATCCATCTTGCTCCAACACCCCAACATCTTCGACGCAGGTGTCGCAGGTCTTCCCGACGATGACGCCGGTGAACTTCCCGCCGCCGTTGTTGTTTTGGAGCACGGAAAGACGATGACGGAAAAAGAGATCGTGGATTACGTCGCCAGTCAAGTAACAACCGCGAAAAAGTTGCGCGGAGGAGTTGTGTTTGTGGACGAAGTACCGAAAGGTCTTACCGGAAAACTCGACGCAAGAAAAATCAGAGAGATCCTCATAAAGGCCAAGAAGGGCGGAAAGATCGCCGTG |  |
