# Supplementary material for: Akaby—Cell-free protein expression system for linear templates
Source: PLoS One. 2022 Apr 7;17(4):e0266272. doi: 10.1371/journal.pone.0266272 (PMC8989226; doi:10.1371/journal.pone.0266272)
Supplement: S2 Table — (DOCX) [file pone.0266272.s007.docx]

**(STable 2) Plasmid information**

| Name | Purpose |
| --- | --- |
| pKDsgRNA-trmI | RecB knockout |
| pKD4 | RecB knockout |
| T7-GFP | eGFP expression in TXTL |
| T7Max-GFP | eGFP expression in TXTL, PCR template for eGFP gene |
| T7Max-Fluc | PCR template for FLuc gene |
